# Comorbidity patterns and health-related quality of life in a cohort of Australian women cancer survivors

**DOI:** 10.1007/s11136-026-04191-2

**Published:** 2026-03-01

**Authors:** Haoyu Zhang, Xue Qin Yu, Michael David, Julie Byles, Mei Ling Yap, Julia Steinberg, Claudia Rutherford, Emily Banks, Karen Canfell, Md Mijanur Rahman

**Affiliations:** 1 School of Public Health, the University of Sydney, Sydney, Australia; 2 The Daffodil Centre, the University of Sydney, Cancer Council NSW, Sydney, Australia; 3 School of Medicine and Dentistry, Griffith University, Gold Coast, Australia; 4 Centre for Women’s Health Research, the University of Newcastle, Newcastle, Australia; 5 Collaboration for Cancer Outcomes Research and Evaluation, South-Western Sydney Clinical School, Ingham Institute for Applied Medical Research, the University of New South Wales, Sydney, Australia; 6 The George Institute for Global Health, the University of New South Wales, Sydney, Australia; 7 Cancer Care Research Unit, Susan Wakil School of Nursing and Midwifery, the University of Sydney, Sydney, Australia; 8 National Centre for Epidemiology and Population Health, Australian National University, Canberra, Australia

**Keywords:** Comorbidity patterns, Women’s health, Health-related quality of life, Short form-36, Cancer survivors

## Abstract

**Purpose:**

This study aimed to identify dominant comorbidity patterns among women cancer survivors and examine how these patterns relate to health-related quality of life (HRQL).

**Methods:**

1544 participants (born 1946–1951) from the Australian Longitudinal Study on Women’s Health diagnosed with cancer during the follow-up period from 1993 to 2019 were included. HRQL is measured with Short Form-36 included in the survey. Latent class analysis was applied to identify comorbidity patterns, and linear regression was used to assess their association with HRQL domains, adjusting for demographic factors.

**Results:**

Five distinct comorbidity classes were identified: relatively healthy (*n* = 880, 57%); hypertension and arthritis (*n* = 278, 18%); arthritis and osteoporosis (*n* = 139, 9%); respiratory conditions (*n* = 170, 11%); and complex multimorbidity (*n* = 93, 6%). Compared to the relatively healthy class, women in all other classes had significantly lower average HRQL (*p* < 0.01). For example, the classes’ adjusted mean score for general health domain varied: relatively healthy (mean = 70.8, reference), hypertension and arthritis (mean = 63.1, 95% CI = 59.9, 66.3), arthritis and osteoporosis (mean = 60.0, 95% CI = 55.8, 64.1), respiratory conditions (mean = 60.9, 95% CI = 57.2, 64.7), and complex multimorbidity (mean = 48.6, 95% CI = 43.4, 53.8). Women in the complex multimorbidity class had the lowest HRQL across all domains: physical functioning [adjusted mean difference from relatively healthy (AMD=− 22.2 and 95% CI − 27.4, − 17.0)], mental health (AMD=-11.4, 95% CI=− 15.4, -7.5).

**Conclusion:**

Comorbidity patterns varied substantially among women cancer survivors and were strongly associated with differences in HRQL. Survivors with complex multimorbidity experienced the greatest impairments. Incorporating comorbidity profiling into survivorship care may help identify high-risk groups and support targeted interventions to optimise quality of life.

**Supplementary Information:**

The online version contains supplementary material available at 10.1007/s11136-026-04191-2.

## Abstract (plain language summary)

More and more people are surviving cancer, but many also live with other long-term health conditions, which can affect their daily life and wellbeing. We carried out this study to better understand how these conditions cluster together and how they impact the quality of life among cancer survivors. Our study looked at whether some health conditions tend to occur together in Australian women cancer survivors, and how these different combinations affect how people feel physically and emotionally. We found that certain patterns of long-term conditions, such as high blood pressure, arthritis, and respiratory diseases, are common among cancer survivors. Women cancer survivors with more complex health issues generally reported lower health-related quality of life across many areas, including general health, bodily pain, physical functioning, and mental wellbeing. These findings suggest that cancer survivors with multiple long-term conditions may need more support to manage their health and improve their quality of life. Health services should consider these patterns when planning care, so survivors get the right help for their unique health needs.

**Supplementary Information:**

The online version contains supplementary material available at 10.1007/s11136-026-04191-2.

## Introduction

The number of people living with cancer (cancer survivors) is growing rapidly, with major contributions from population ageing, advances in early detection, and improvement in cancer treatment [1]. In Australia alone, on average, 463 new cancer cases were diagnosed daily in 2024 [2]. Cancer survivors tend to experience multiple morbidities, with many having pre-existing conditions or being diagnosed with new conditions after the cancer diagnosis [3]. Previous studies report that comorbid conditions among cancer survivors are associated with higher mortality [4], increased health services utilisation [5], reduced physical functioning status, and poorer quality of life [6–8].

Health-related quality of life (HRQL) is a multidimensional construct encompassing perceptions of both positive and negative aspects of physical, social, and psychological functioning and other symptoms produced by a disease or its treatment [9]. It is an important outcome in oncology and cancer epidemiology research. Compared to people without comorbidities, cancer survivors who were diagnosed with two or more comorbidities have significantly lower HRQL [7]. The complex interconnected aetiology across various conditions along with the experience of multiple illnesses, poses significant challenges to traditional single-disease-focused survivorship care approaches.

Current HRQL analyses often simplify multimorbidity by focusing on either the number of conditions or predetermined disease combinations [7, 8]. Such classifications may limit our understanding by focusing only on clinically defined, body-system-specific disease groupings, rather than adopting a more holistic approach that captures the full complexity of an individual’s comorbidity profile. Moreover, conventional approaches often assume that comorbidity patterns are consistent across populations, despite substantial variation in the distribution of chronic conditions by demographic and socio-economic characteristics [10]. Unlike comorbidity indices that collapse disease burden into a single aggregated score [11], pattern-based multimorbidity approaches preserve the heterogeneity of disease co-occurrence by identifying distinct constellations of conditions [12]. Compared with index- or comorbidity count-based approaches, pattern-based analyses therefore have greater potential to generate person-centred insights into primary care needs following a cancer diagnosis. To date, multimorbidity clustering has not been applied specifically to women cancer survivors in Australia.

Understanding the complex comorbidity patterns among cancer survivors requires an appropriate analytical approach. Latent class analysis (LCA) is a data driven approach that identifies latent subgroups and assumes a finite number of classes within a population [11] and has demonstrated its superior utility in multimorbidity clustering [13]. Compared to other clustering methodologies, LCA explicitly accounts for measurement error and provides probabilistic class membership, allowing for more accurate identification or clinically meaningful subgroups [11, 13]. Leveraging this method, we aimed to (1) identify the comorbidity patterns among a cohort of Australian women cancer survivors, and (2) examine how the identified patterns were associated with HRQL among cancer survivors.

## Methods

### Data source and sample

This study used data from the Australian Longitudinal Study on Women’s Health (ALSWH) between 1996 and 2022 and the linked Australian Cancer Database between 1993 and 2019. The ALSWH is a large, population-based survey that includes four cohorts of Australian women born in different periods, with over 57,000 participants [14]. This study is based on the birth cohort 1946–1951, aged 45–50 in 1996. At baseline, participants were randomly selected from a Medicare database with intentional oversampling of women from rural and remote areas at twice the rate of urban women to capture variation in health experiences beyond metropolitan regions [14]. In total, 13,714 participants self-completed a postal questionnaire at baseline (response rate of 52–56%). These participants were first followed up in 1998, with follow-ups every three years thereafter until 2022 when the 9th was completed (77% eligible). The ALSWH survey was designed to cover women’s physical and mental health, health behaviours, reproductive health, and social factors questionnaire data. The survey data have been linked to cancer diagnosis through the Australian Cancer Database. Details about the ALSWH surveys have been published elsewhere [15].

The study included 1544 women with a cancer diagnosis, ascertained via data linkage with the Australian Cancer Database from 1993 to 2019 who completed an ALSWH survey within three years of the diagnosis (Supplementary Fig. 1). Only primary invasive cancer diagnoses were identified as eligible cases (Supplementary Table 1). Non-melanoma skin cancer was not eligible and not usually recorded in the cancer database [16]. Participants diagnosed with cancer three years before the return date of the baseline survey (*n* = 278), and those who did not complete a survey within three years of the recorded diagnosis (*n* = 885) were excluded from the study. For each woman with cancer, data from the immediate survey completed after her cancer diagnosis (within three years) were used for this analysis.

### Health-related quality of life

HRQL was measured using the Medical Outcomes Study Short Form-36 (SF-36) as administered in the ALSWH survey [17], which consists of 36 questions encompassing eight domains: physical functioning, social functioning, mental health, general health, vitality, bodily pain, role emotional, and role physical. Raw scores were standardised into a score out of 100, formula [(Raw score-minimum possible raw score/possible raw score range)×100], with higher scores indicating better outcomes. Role emotional, role physical, and social functioning domains were not included in the current analysis because the derived scores were categorical and non-normal (Supplementary Fig. 2) [14, 15, 17, 18].

### Comorbidity

Comorbidities were based on self-reports of doctor-diagnosis (by either a general practitioner or a specialist) of 9 major conditions: arthritis (osteoarthritis and rheumatoid arthritis), asthma, bronchitis (chronic bronchitis, chronic emphysema, and chronic obstructive pulmonary disease), diabetes mellitus (type 1, type 2, and prediabetes), heart disease (myocardial infarction, angina, arrhythmias, and heart failure), hypertension, osteoporosis, stroke, and thrombosis. Other comorbidities (e.g. mental health conditions, ocular conditions, hepatitis, renal diseases, polycystic ovary syndrome, and neurodegenerative diseases) were not included due to incomplete data or being inconsistently included across survey waves [19, 20].

At baseline, respondents were asked whether they had ever been diagnosed by a doctor with each listed chronic condition (Yes, No). At each follow-up, respondents were asked whether they had been newly diagnosed with the conditions since the previous follow-up (Yes, No). The conditions were considered enduring, as individuals typically live with these diagnoses for the remainder of their lives, experiencing prolonged effects on their well-being and quality of life. A person was considered to have a cumulative history of the conditions if she had reported a comorbidity diagnosis at her index survey or in any previous ones.

### Socio-demographic and behavioural factors

Sociodemographic characteristics were derived from the ALSWH survey, including age, country of birth (Australia, other English-speaking countries, or all other countries), area of residence (major cities, inner or outer regional, remote or very remote), highest educational qualification (schooling up to 12 years or lower, qualification/diploma/certificate or equivalent, university degree or higher), and difficulty in managing available income (easy/not too bad, difficult, impossible). Behavioural factors were also from the ALSWH questionnaire and included body mass index (underweight and normal weight < 25, overweight ≥ 25.0 and < 30.0, and obese ≥ 30.0), and smoking status (never smoker, former smoker, and current smoker).

### Statistical analysis

The distribution of cancer cases by socio-demographic factors and health characteristics was explored via descriptive statistics. The observed pair-wise comorbidity patterns were presented as a two-by-two contingency heatmap to assess any potential dependence between the conditions. LCA was conducted to identify comorbidity patterns among cancer survivors [11], with each comorbidity included as an indicator variable. The presence or absence of each condition was used to determine latent (unobserved) classes based on their probability (prevalence) distributions. Every participant was assigned to a class based on her highest posterior probability of belonging to that class. Models with two to seven classes were fitted and evaluated for model selection. The optimal number of classes was determined based on the Akaike information criterion, the Bayesian information criterion, entropy, and interpretability of the classes. The detailed model selection procedure, along with model fit statistics are included in the supplementary materials (Supplementary Tables 2–4). Identified classes were labelled according to the two most prevalent conditions within each class or, where appropriate, by overall comorbidity burden. The LCA classes were explored by demographics, behavioural, and health characteristics. Chi-squared tests and Kruskal-Wallis tests were performed to assess differences between latent classes for the categorical variables and age at diagnosis, respectively. The obtained classes were plotted by condition prevalence and labelled according to their patterns.

To assess the heterogeneity of HRQL across the latent classes, we first took a non-modelling approach. Each participant’s HRQL score was compared with the ALSWH population median score from her index survey at cancer diagnosis. Scores below the median were labelled as “low HRQL”, for each domain respectively. Second, multivariable linear regression models were performed for each domain score, adjusting for age, marital status, difficulty in managing available income, education qualifications, country of birth, and remoteness of residence. Adjusted mean difference (AMD) based on least squares means and its 95% confidence interval (CI) were estimated from each domain-specific model. Missing values for covariates used in the regression were initially imputed with survey answers from either the baseline (country of birth, ability to manage available income) or the subsequent surveys (highest education) if appropriate and available. Multiple imputations were performed on demographic characteristics for the remainder of missingness. Details regarding the statistical strategy for handling missingness is described in the Supplementary Materials. All analyses were performed in R version 4.4.2.

## Results

**Fig. 1:**
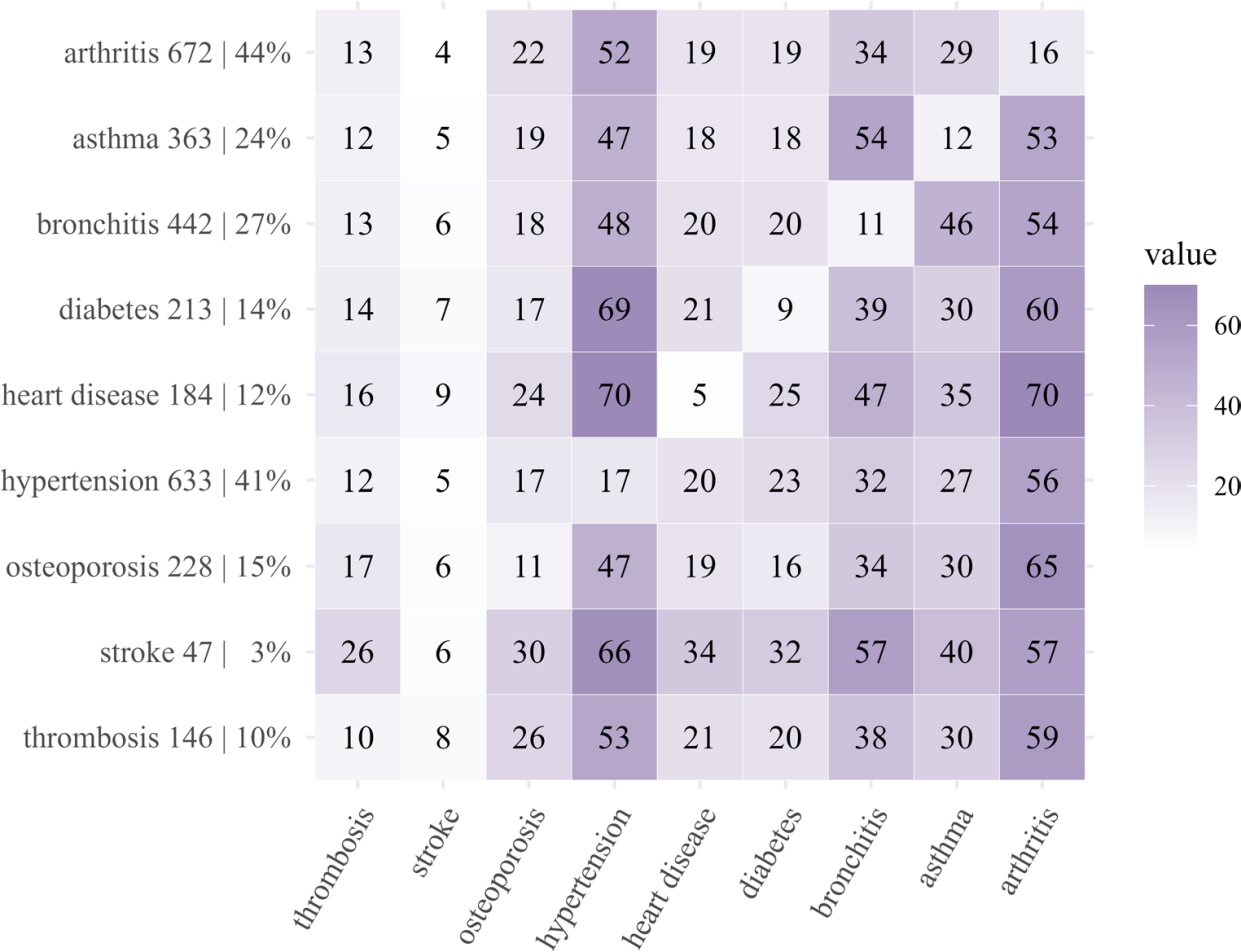
Contingency heatmap of chronic condition distribution and crude percentage co-occurrence among women cancer survivors

Of 1544 women included in the study, 700 (45%) had been diagnosed with breast cancer, 227 (15%) melanoma, 153 (10%) digestive system cancer, 135 (9%) gynaecological cancer, and 329 (21%) with all other cancer types (excluding non-melanoma skin cancers). The median age at cancer diagnosis was 60.8 years, with an interquartile range (IQR) of 53.8–66.4. Approximately, one out of five women who were diagnosed with cancer did not report any comorbidity (22%), and over half (54%) reported two or more comorbidities (Table 1). Arthritis (44%), hypertension (41%), and bronchitis (27%) were the most prevalent conditions (Fig. 1). Hypertension was particularly common among women with heart disease (70%) and diabetes (69%). Bronchitis and asthma had a higher co-occurrence rate of 54%.

**Table 1:** Characteristics of participating women cancer survivors at time of diagnosis by latent class membership

Variable	Total^*^	Latent Classes^*^				
		1	2	3	4	5
N (%)	1544	880 (57.0)	274 (17.7)	135 (8.7)	169 (10.9)	86 (5.6)
Age at diagnosis †, median (IQR)	60.8 (53.8–66.4)	57.6 (51.1–63.8)	64.6 (59.5–68.2)	64.9 (59.4–67.9)	61.8 (53.6–66.5)	66.1 (60.7–68.8)
Cancer Site, n (%)						
Breast	700 (45.3)	407 (46.2)	125 (45.6)	63 (46.7)	75 (44.4)	30 (34.9)
Melanoma	227 (14.7)	131 (14.9)	35 (12.8)	24 (17.8)	22 (13.0)	15 (17.4)
Digestive	153 (9.9)	93 (10.6)	28 (10.2)	11 (8.1)	14 (8.3)	7 (8.1)
Gynaecological	135 (8.7)	79 (9.0)	27 (9.9)	7 (5.2)	15 (9.5)	6 (7.0)
All other cancer^**^	329 (21.3)	170 (19.3)	59 (21.5)	30 (22.2)	43 (25.4)	28 (32.6)
Number of Comorbidities, n (%)						
0–1	–	715 (81.2)	0	0	0	0
2–3	–	165 (18.8)	232 (84.7)	92 (68.1)	92 (54.4)	< 10 (< 10%)
≥ 4	–	0	42 (15.3)	43 (31.9)	77 (45.6)	> 70 (> 90%)
Marital Status †, n (%)						
Married/De facto	1160 (75.8)	687 (78.7)	207 (76.8)	98 (73.7)	117 (69.6)	51 (59.3)
Never married/divorced/ separated/widowed	370 (24.2)	186 (21.3)	63 (23.3)	35 (26.3)	51 (30.4)	35 (40.7)
Difficulty to manage income †, n (%)						
Easy/not too bad	987 (64.7)	604 (69.7)	168 (61.8)	89 (66.4)	95 (56.5)	31 (36.5)
Difficult	361 (23.7)	186 (21.5)	69 (25.4)	29 (21.6)	44 (26.2)	33 (38.8)
Impossible	178 (11.7)	77 (8.9)	35 (12.9)	16 (11.9)	29 (17.3)	21 (24.7)
Education, n (%)						
12 years or less	984 (63.9)	548 (62.5)	184 (67.2)	83 (61.5)	105 (62.1)	64 (74.4)
Certificate/Diploma or Equivalent	337 (21.9)	190 (21.7)	58 (21.2)	30 (22.2)	45 (26.6)	14 (16.3)
University or higher	220 (14.3)	139 (15.8)	32 (11.7)	22 (16.3)	19 (11.2)	8 (9.3)
Area of Residence †, n (%)						
Major cities	595 (38.6)	369 (42.0)	84 (30.7)	48 (35.6)	64 (37.9)	30 (34.9)
Inner regional	623 (40.4)	320 (36.4)	132 (48.2)	61 (45.2)	71 (42.0)	39 (45.3)
Remote/outer regional	324 (21.0)	189 (21.5)	58 (21.2)	26 (19.3)	34 (20.1)	17 (19.8)
Country of Birth †, n (%)						
Australia	1195 (78.1)	658 (75.4)	218 (80.1)	107 (81.1)	147 (87.5)	65 (75.6)
Other English-speaking countries	209 (13.7)	133 (15.2)	37 (13.6)	15 (11.4)	14 (8.3)	10 (11.6)
All other countries	127 (8.3)	82 (9.4)	17 (6.2)	10 (7.6)	7 (4.2)	11 (12.8)
Body Mass Index †, n (%)						
Underweight and Healthy (< 25)	584 (39.5)	390 (46.2)	59 (22.6)	56 (23.7)	61 (38.6)	18 (21.4)
Overweight (≥ 25 and < 30)	467 (31.6)	283 (33.5)	71 (27.2)	45 (35.2)	42 (26.6)	26 (31.0)
Obese (≥ 30)	425 (28.8)	172 (20.4)	131 (50.2)	27 (21.1)	55 (34.8)	40 (47.6)
Smoking Status †, n (%)						
Never smoked	921 (60.2)	536 (61.5)	179 (65.8)	82 (61.2)	79 (47.3)	45 (52.3)
Ex-smoker	486 (31.7)	270 (31.0)	82 (30.1)	42 (31.3)	60 (35.9)	32 (37.2)
Current smoker	124 (8.1)	66 (7.6)	11 (4.0)	10 (7.5)	28 (16.8)	9 (10.5)

IQR, interquartile range; - Cells suppressed to maintain confidentiality

Class 1 Relatively healthy; Class 2 hypertension and arthritis; Class 3 arthritis and osteoporosis.

Class 4 respiratory conditions; Class 5 higher comorbidity.

† *p* < 0.05 for Chi-squared test for categorical variable, or Kruskal-Wallis test for age, between latent classes

* Only complete cases are reported. The frequencies in some variables may not add up to the total sample size due to missingness

** Excluding non-melanoma skin cancer of any kind

* Numbers in each tile represent the cross-tabulation percentages between the two conditions. For instance, arthritis-thrombosis 13% is interpreted as: among women who had arthritis, 13% had thrombosis

** Numbers in the diagonal tiles (bottom left to top right) refer to percentage of women who had the respective conditions and none other. For instance, arthritis-arthritis 16% is interpreted as: among women who had arthritis, 16% of them are free from any other conditions (they had cancer in the first place)

95%CI, 95% confidence interval. AMD is calculated based on least squares means

General health, *n* = 1506, % of missingness = 2.46%; Physical function, *n* = 1535, % of missingness = 0.58%

Mental health, *n* = 1537, % of missingness = 0.45%; Bodily pain, *n* = 1540, % of missingness = 0.25%

Vitality, *n* = 1536, % of missingness = 0.52%

**Fig. 2:**
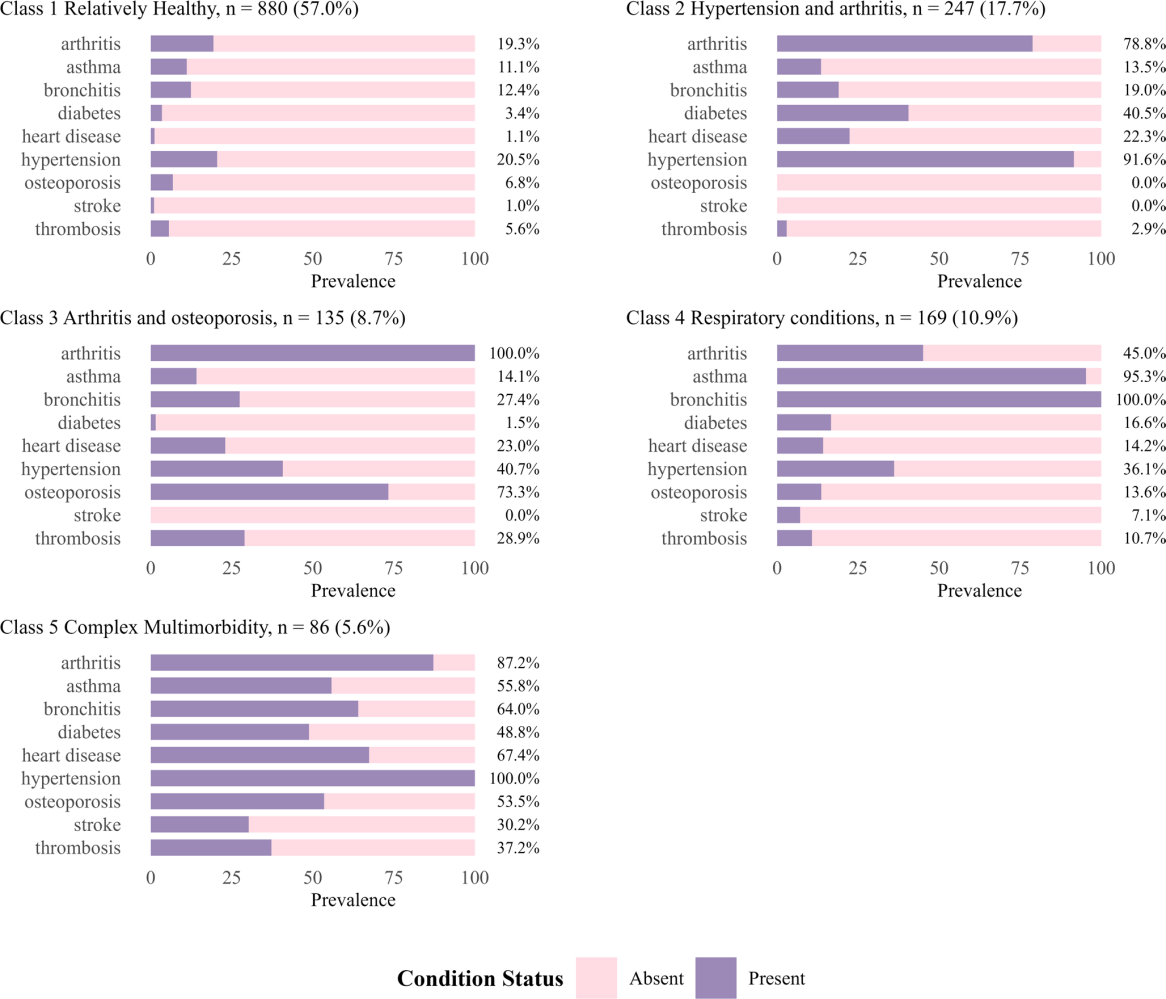
Crude comorbidity prevalence distribution by latent class membership

**Fig. 3:**
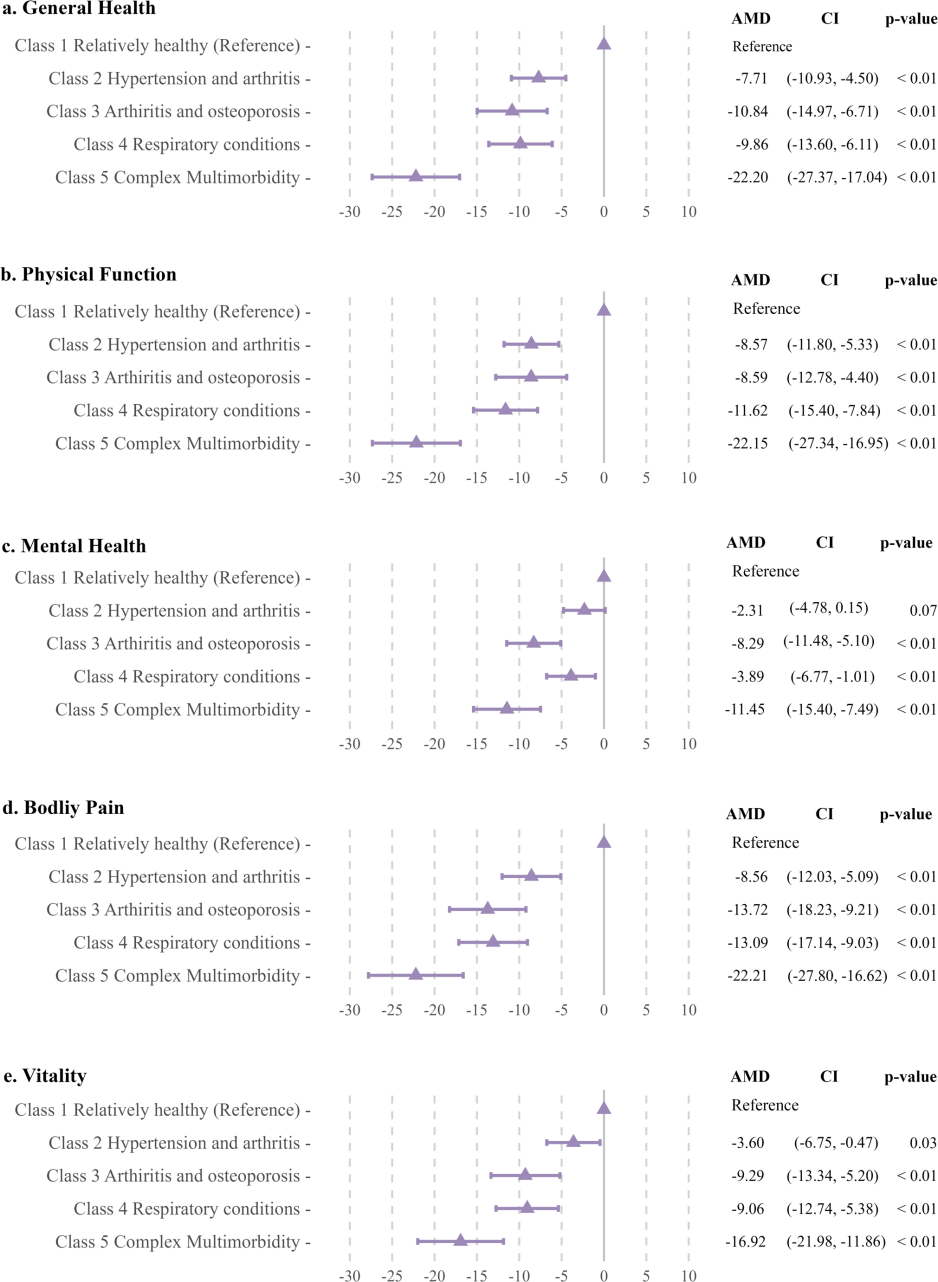
Adjusted mean difference (AMD) and 95%CI for health-related quality of life domains by latent classes, compared to the relatively health class, adjusted for age, marital status, difficulty in managing available income, education qualifications, country of birth, and region of residence

Considering the fit statistics (Supplementary Tables 2–4) and interpretability of the classes, a 5-class model was considered the optimum model. The five classes obtained from the LCA analysis were presented by demographic characteristics (Table 1 and Supplementary Table 5), health characteristics (Fig. 2) and HRQL domain distribution and scores (Fig. 3, Supplementary Figs. 2 and 3). Based on their comorbidity prevalence patterns, the classes were labelled: 1 *relatively healthy*, 2 *hypertension and arthritis*, 3 *arthritis and osteoporosis*, 4 *respiratory conditions*, and 5 *complex multimorbidity*. The latent classes varied in size, with class 1: 57% (*n* = 880), class 2: 18% (*n* = 274), class 3: 9% (*n* = 135), class 4: 11% (*n* = 169), and class 5: 6% (*n* = 86).

Each class featured a distinctive multimorbidity pattern (Fig. 2). Hypertension and arthritis were presented in all classes, reflecting the prevalence of these conditions among women at this life stage. Around one in five participants in the *relatively healthy* class had hypertension (21%) or arthritis (19%), but they had a low prevalence of other comorbidities. In the *hypertension and arthritis* class, hypertension (92%) and arthritis (79%) were the most prevalent conditions, affecting most women in this class. All members of the *arthritis and osteoporosis* class had arthritis, and 73% also had osteoporosis. Over 40% of all patients with osteoporosis were assigned to this class. Bronchitis and asthma were the predominant conditions in the *respiratory conditions* class, with prevalence rates of 100% and 95%, respectively. The *complex multimorbidity* class had a notably high prevalence of multiple chronic conditions across different body systems, and all reported hypertension (100%). Conditions such as arthritis (87%) and bronchitis (64%) also showed high prevalence in this class, reinforcing clustering of severe comorbidities.

The participants’ demographic, behavioural, and clinical characteristics by LCA classes are presented in Table 1. Women in the *complex multimorbidity* class had the highest median age at cancer diagnosis (66.1) while the *relatively healthy* class was the youngest group (median age 57.6). Over 80% of women in the *complex multimorbidity* class had five or more co-existing conditions. Approximately 60% of women in this class reported finding it difficult or impossible to manage their income and 78% were overweight or obese. Obesity was also common among women in the *hypertension and arthritis* class (50% obese). Participants in the *respiratory conditions* class were more likely to be a current or ex-smoker compared to the other classes. Notably, the distribution of cancer site does not differ significantly across latent classes (Supplementary Table 5). The distribution of HRQL scores within each class is described in Supplementary Fig. 3. In all classes, more than half of the survivors had lower scores than the median observed among ALSWH participants in their index survey wave. The majority of women in the *complex multimorbidity* class scored poorly on general health (88%) and physical functioning (88%) subscales, lower than the population median. 73% of women in the arthritis and osteoporosis class had lower than median mental health scores, and 75% of them had low vitality scores. Figure 3 presents the results (AMD and 95%CI) of multivariable linear regression models for each HRQL domain by LCA classes. Compared to the women in the *relatively healthy* class, those in most other classes reported significantly lower scores in almost all domains (*p* < 0.01). Members in the *complex multimorbidity* class reported the lowest scores across all domains. Specifically in general health (AMD = − 22.2, CI = − 27.4 – − 17.0), physical function (AMD = − 22.2, CI = − 27.2 – − 17.0), mental health (AMD = − 11.45, CI = − 15.4 – − 7.5), bodily pain (AMD = − 22.2, CI = − 27.8 – − 16.6), and vitality (AMD = − 16.9, CI = − 22.0 – − 11.9). The *arthritis and osteoporosis* class exhibited the second lowest scores in general health (AMD = − 10.84, CI = − 14.97 – − 6.71), mental health (AMD = − 8.29, CI = − 11.48 – − 5.10), bodily pain (AMD = − 13.72, CI = − 18.23 – − 9.21), and vitality (AMD = − 9.29, CI = − 13.34 – − 5.20). On the other hand, women in the *respiratory conditions* class reported the second lowest physical function scores (AMD = − 11.62, CI = − 15.40 – − 7.84).

## Discussion

This study investigated comorbidity patterns among women cancer survivors and how these patterns related to their HRQL within the first few years after cancer diagnosis. We identified five subgroups of cancer survivors with distinct comorbidity patterns that differentially associated with varying HRQL domains. Compared to the *relatively healthy* class, women in all other groups reported significantly lower HRQL, with the *complex multimorbidity* group reporting the lowest HRQL scores across all domains. These findings highlight the heterogeneity among cancer survivors, with each class characterised by specific comorbidity patterns, demographics, and behavioural factors, which were differentially associated with their HRQL across domains.

The observed comorbidity patterns are highly dependent on population characteristics. Our findings may not be directly comparable to previous studies due to variations in the comorbidity spectrum and count, methodological differences, study population, and baseline demographic characteristics and health conditions. However, the classification derived from our model is highly consistent with previous cluster analysis of multimorbidity patterns in general populations [21, 22] and agree with clinically meaningful groupings [23]. An exploratory factor analysis of an Australian working population revealed disease clusters of musculoskeletal conditions, metabolic-obesity conditions, asthma and chronic obstructive pulmonary disease (COPD), corresponding to the *arthritis and osteoporosis* group, the *hypertension and arthritis* group, and the *respiratory conditions* group in our model, respectively [21]. The *complex multimorbidity* class represents the minority of cancer survivors who experience multiple chronic conditions, in addition to cancer.

The LCA classes observed in our study suggest that some chronic conditions may aggregate due to their shared risk and aetiological factors, similar phenotypic expressions, or some conditions that serve as mutual risk factors for each other. For example, hypertension, arthritis, and diabetes mellitus were the most common conditions among the members in the *hypertension and arthritis* class. Obesity is a well-established cause of both hypertension and diabetes [24], and these three health burdens have a similar epidemiological distribution [25]. Furthermore, multiple studies revealed the association between obesity and arthritis [26, 27], the second most prevalent condition in this class. We believe that obesity acts as a latent variable linking the comorbidity pattern.

The *arthritis and osteoporosis* class had a 100% prevalence of arthritis, and 73% prevalence of osteoporosis. Although our data source did not differentiate between rheumatoid arthritis (RA) and osteoarthritis (OA), their intertwined relationships with osteoporosis provide meaningful insights. Despite their distinct aetiologies, degenerative physical function has a bidirectional correlation with both OA and osteoporosis [28, 29], and osteoporosis can be a common complication of RA [30, 31], which may help explain the high likelihood of concurrent conditions. Compared with the hypertension and arthritis class, women in this group may experience higher healthcare utilisation, given that hypertension and arthritis are commonly diagnosed in primary care, while osteoporosis usually requires referral to medical imaging for diagnosis.

Almost all members in the *respiratory conditions* class had asthma and chronic bronchitis/emphysema/COPD simultaneously. While debates continue over the pathology underlying the co-occurrence, the term asthma-COPD overlap is used to describe their clinical phenotype, highlighting the burden associated with this comorbidity pattern [32, 33]. More than half of the women in this class reported either former or current smoking, a pattern that contrasts with the smoking status distribution observed in the other classes [34]. Numerous studies have documented the association between smoking and respiratory conditions, such as asthma and COPD [32, 35, 36]. In Australia, smoking is the leading cause of COPD, contributing to a significant number of cases [37]. Cancer survivors in the *complex multimorbidity* class demonstrated a complex pattern that spanned cardiovascular, musculoskeletal, metabolic, and pulmonary conditions, manifesting the age-related accumulation of multimorbidity. Lack of marital support and poor social determinants of health in this subgroup highlight the need for extensive social support and healthcare resources [10].

Regarding the association between LCA classes and HRQL domains, the *relatively healthy* class was set as the reference group, as nearly all women in this class had fewer than two conditions, with 38% free of any comorbidity. The *complex multimorbidity* class were notably older than those in other classes. Age-related differences were considered because older age is associated with lower HRQL and contributes significantly to many health conditions included in our model. However, when comparing the HRQL scores of members with age-specific, index wave-specific medians, a substantial proportion still reported scores below the norm, further supporting our findings. This indicates the need for cancer survivorship care attention to those who have multiple comorbidities, are elderly, and are having difficulty managing income. Women in the *arthritis and osteoporosis* class reported the second-lowest HRQL scores in the general health, mental health, bodily pain, and vitality domains. Osteoarthritis and rheumatoid arthritis, as the leading comorbidities in this class, cause pain and stiffness, limiting movement and deteriorating physical function. Having rheumatic disease was associated with worse HRQL due to worsened pain perception, movement limitation, thereby supporting our findings [38]. While the deficits in HRQL in the *hypertension and arthritis* class were less severe compared to more severe comorbidity classes, the presence of both hypertension and arthritis were associated with significant reductions in physical function and bodily pain domains. The high prevalence of obesity in this group raises concerns about its association with joint stress, cardiovascular risk, and other long-term health outcomes [39].

Although the interclass differences were significant, the latent classes were less strongly related to mental health when compared to other domains. We suspect this is due to the selection of our reference group, a *relatively healthy* class from our LCA model. A growing body of evidence suggests that mental health tends to improve with age, with older adults often reporting lower levels of psychological distress and greater emotional well-being than younger individuals [40]. Simultaneously, results from our study suggests that older people tend to experience more comorbidities. Therefore, the interplay between age, comorbidity, and mental health may offset the variation across classes. Comparisons made among cancer survivors may also contribute to the seemingly reduced impact of comorbidity on the mental health domain.

In addition, we found that difficulty in managing available income is a significant predictor, even after adjustment in our regression model. Women who reported having difficulty or finding it almost impossible to manage available income had significantly lower HRQL scores across all domains, adjusting for latent classes and other demographic variables. Behavioural factors, such as body mass index, smoking status, and physical exercise, were not included as adjustments in the regression model due to potential bidirectional correlations between these factors and certain HRQL domains [41, 42].

A key strength of our study is the use of data from a large cohort with over 20 years of follow-up, spanning ages 45–50 to 68–73. The cohort broadly represents the general Australian women population in this age group, ensuring the generalizability of our findings to this population. We assessed HRQL using the well-validated SF-36 questionnaire, which assess various HRQL domains [17, 43]. Most prior studies examining the correlation between multimorbidity patterns and HRQL have relied on non-model approaches, including analysis of individual conditions, pairwise combinations, comorbidity counts, or pre-determined comorbidity groups [7, 40, 44, 45]. In recent years, researchers have employed statistical modelling methods such as cluster analysis [46, 47], factor analysis [21], and LCA [48, 49]; however, none have linked these classifications to HRQL. This study is the first to apply LCA to examine the relationship between multimorbidity patterns and HRQL in women cancer patients, leveraging extensive longitudinal data. LCA surpasses conventional clustering methods by using data-driven, probability-based classifications that determine the optimal number of classes through various diagnostic tests [13]. Our study suggests that distinct multimorbidity patterns among cancer survivors may capture differences in care needs and mechanisms underlying variation in HRQL that are not well represented by aggregate comorbidity measures. These findings provide a basis for future work to evaluate whether such patterns can be used to better target survivorship interventions and align follow-up care with patient need.

Our study has some limitations. First, the comorbidities were ascertained from self-reported doctor-diagnosed conditions. A previous study of this cohort found that participants tend to underreport or overreport their medical history, depending on the condition, making it difficult to predict the direction of bias [20]. Second, our study utilised comorbidity information up to the survey wave, which occurred up to three years after cancer diagnosis. Individuals who were lost to follow-up due to deteriorating health may have been excluded, introducing survivor bias that could have led to an overestimation of overall HRQL, but also impact prevalence estimates for different classes. Response bias may also lead to some subgroups with specific severe comorbidities being present in the general population but absent from the study data. Furthermore, by limiting the observation period to three years post-diagnosis, potential long-term interactions between cancer, anti-cancer treatments, and the selected comorbid conditions were not captured. Third, conditions included in our study were constrained by the ALSWH survey design. Changes made in this survey over time as the cohort progressed may lead to variation in the comorbidity list across different survey waves. However, this may not affect the comorbidity patterns observed in our study as all the predictors included were consistent across the ALSWH survey, and the comparisons were made between groups within the cohort, which are less prone to bias than other analyses. Given the retrospective nature of our variable, severity information cannot be incorporated in our study either. Other limitations regarding the ALSWH survey have been discussed elsewhere [14, 15, 20]. Fourth, our study offers insights into comorbidity patterns among women cancer survivors. However, the generalisability and potential transferability of these findings require further investigation. Finally, our study focused on all cancers combined. We could not conduct cancer-type specific analysis as LCA requires a sufficient sample size, which was not available for most cancer types included in the study. Additionally, we could not account for cancer stage at diagnosis and treatment modality (which are absent in our data) that may confound HRQL research. Future studies could incorporate these aspects and explore the clinical implications of our findings related to comorbidity patterns and HRQL.

## Conclusion

We identified five distinct comorbidity patterns among women cancer survivors. The groups have distinct distributions in demographic and behavioural factors. HRQL differed significantly across women with different comorbidity patterns. Most women were in the *relatively health* class, with the highest levels of HRQL. Around 1 in 17 were in the *complex multimorbidity* group and had the lowest scores in all HRQL domains. The arthritis and osteoporosis group also faced notable deficits in multiple domains, while the respiratory conditions group had the second lowest physical health scores. These findings emphasise positive outcomes for most survivors and the need to consider comorbidity in comprehensive and person-centred survivorship care plans for women cancer survivors to support their HRQL.

## Supplementary Information

Below is the link to the electronic supplementary material.

Supplementary Material 1

## Data Availability

This data analysis was conducted under conditions approved by the relevant ethics committee(s). As a condition of approval, data are not shareable. Access to data by other individuals or agencies would require appropriate ethical approvals to be in place.

## References

[1] Sung, H., Ferlay, J., Siegel, R. L., Laversanne, M., Soerjomataram, I., Jemal, A., et al. (2021). Global cancer statistics 2020: GLOBOCAN estimates of incidence and mortality worldwide for 36 cancers in 185 countries. *C Ca: A Cancer Journal for Clinicians*. 10.3322/caac.21660 10.3322/caac.21660 33538338

[2] Australian Institute of Health and Welfare. Cancer data in Australia [Internet]. Canberra: AIHW (2024). [cited 2024 Aug 4]. Available from: https://www.aihw.gov.au/reports/cancer/cancer-data-in-australia/contents/overview

[3] Joshy, G., Thandrayen, J., Koczwara, B., Butow, P., Laidsaar-Powell, R., Rankin, N., et al. (2020). Disability, psychological distress, and quality of life in relation to cancer diagnosis and type: population-based Australian study of 22,505 cancer survivors and 244,000 people without cancer. *Bmc Medicine*. 10.1186/s12916-020-01830-4 33256726 10.1186/s12916-020-01830-4 PMC7708114

[4] Wallace, E., Salisbury, C., Guthrie, B., Lewis, C., Fahey, T., & Smith, S. M. (2015). Managing patients with Multimorbidity in primary care. *Bmj*. 10.1136/bmj.h176 25646760 10.1136/bmj.h176

[5] Lehnert, T., Heider, D., Leicht, H., Heinrich, S., Corrieri, S., Luppa, M., et al. (2011). Healthcare utilization and costs of elderly persons with multiple chronic conditions: A review. *Med Care Res Rev*. 10.1177/1077558711399580 21813576 10.1177/1077558711399580

[6] Fortin, M., Lapointe, L., Hudon, C., Vanasse, A., Ntetu, A. L., & Maltais, D. (2004). Multimorbidity and quality of life in primary care: A systematic review. *Health and Quality of Life Outcomes*. 10.1186/1477-7525-2-51 15380021 10.1186/1477-7525-2-51 PMC526383

[7] Fortin, M., Bravo, G., Hudon, C., Lapointe, L., Almirall, J., Dubois, M. F., et al. (2006). Relationship between Multimorbidity and health-related quality of life in primary care patients. *Quality of Life Research*. 10.1007/s11136-005-8661-z 16411033 10.1007/s11136-005-8661-z

[8] Fu, M. R., Axelrod, D., Guth, A. A., Cleland, C. M., Ryan, C. E., Weaver, K. R., et al. (2015). Comorbidities and quality of life among breast cancer survivors: A prospective study. *Journal of Personalized Medicine*10.3390/jpm5030229 26132751 10.3390/jpm5030229 PMC4600145

[9] Osoba, D. (1994). Lessons learned from measuring health-related quality of life in oncology. *Journal of Clinical Oncology*. 10.1200/JCO.1994.12.3.608 8120561 10.1200/JCO.1994.12.3.608

[10] Pathirana, T. I., & Jackson, C. A. (2018). Socioeconomic status and multimorbidity: A systematic review and meta-analysis. *Australian and New Zealand Journal of Public Health*. 10.1111/1753-6405.12762 29442409 10.1111/1753-6405.12762

[11] Lazarsfeld, P. F., & Henry, N. W. (1968). *Latent structure analysis*. Houghton Mifflin.

[12] Kongsted, A., & Nielsen, A. M. (2017). Latent class analysis in health research. *Journal of Physiotherapy*. 10.1016/j.jphys.2016.05.018 27914733 10.1016/j.jphys.2016.05.018

[13] Nichols, L., Taverner, T., Crowe, F., Richardson, S., Yau, C., Kiddle, S., et al. (2022). In simulated data and health records, latent class analysis was the optimum Multimorbidity clustering algorithm. *Journal of Clinical Epidemiology*, *152*, 164–175. 10.1016/j.jclinepi.2022.10.011 36228971 10.1016/j.jclinepi.2022.10.011 PMC7613854

[14] Lee, C., Dobson, A. J., Brown, W. J., Bryson, L., Byles, J., Warner-Smith, P., et al. (2005). Cohort profile: The Australian longitudinal study on women’s health. *International Journal of Epidemiology*. 10.1093/ije/dyi098 15894591 10.1093/ije/dyi098

[15] Dobson, A. J., Hockey, R., Brown, W. J., Byles, J. E., Loxton, D. J., McLaughlin, D., et al. (2015). Cohort profile update: Australian longitudinal study on women’s health. *International Journal of Epidemiology*. 10.1093/ije/dyv110 26130741 10.1093/ije/dyv110

[16] Marzorati, C., Riva, S., & Pravettoni, G. (2017). Who is a cancer survivor? A systematic review of published definitions. *Journal of Cancer Education*. 10.1007/s13187-016-0997-2 26854084 10.1007/s13187-016-0997-2

[17] McHorney, C. A., Ware, J. E. Jr, & Raczek, A. E. (1993). The MOS 36-Item Short-Form health survey (SF-36): II. Psychometric and clinical tests of validity in measuring physical and mental health constructs. *Medical Care*. 10.1097/00005650-199303000-00006 8450681 10.1097/00005650-199303000-00006

[18] Stevenson, C. (1996). SF-36: Interim norms for Australian data. In: Australian Institute of Health and Welfare. Health-related quality of life measurement. Canberra: AIHW; https://www.aihw.gov.au/reports/health-welfare/health-measurement-data. Accessed 4 Jun 2025.

[19] Byles, J., Hockey, R., McLaughlin, D., Dobson, A., Brown, W., Loxton, D. (2015). Chronic conditions, physical function, and health care use. In: Australian Longitudinal Study on Women’s Health: Annual report 2015. Australian Government Department of Health; https://www.alswh.org.au/publications/879-chronic-conditions-health-care-use Accessed Jan 2025.

[20] Dobson, A., Forder, P., Hockey, R., Egan, N., Cavenagh, D., Waller, M. (2020). May. Report prepared for the Australian Government Department of Health. In: Australian Longitudinal Study on Women’s Health: Major Report Series. https://www.alswh.org.au/publications/1284-major-report-may-2020. Accessed 4 Jun 2025.

[21] Holden, L., Scuffham, P. A., Hilton, M. F., Muspratt, A., Ng, S. K., & Whiteford, H. A. (2011). Patterns of Multimorbidity in working Australians. *Population Health Metrics*. 10.1186/1478-7954-9-15 21635787 10.1186/1478-7954-9-15 PMC3123553

[22] Cornell, J. E., Pugh, J. A., Williams, J. W. Jr, Kazis, L., Lee, A. F., Parchman, M. L., et al. (2008). Multimorbidity clusters: Clustering binary data from a large administrative medical database. Applied Multivariate Research. 10.22329/amr.v12i3.658

[23] Prados-Torres, A., Calderón-Larrañaga, A., Hancco-Saavedra, J., Poblador-Plou, B., & van den Akker, M. (2014). Multimorbidity patterns: A systematic review. *Journal of Clinical Epidemiology*. 10.1016/j.jclinepi.2013.09.021 24472295 10.1016/j.jclinepi.2013.09.021

[24] Patel, S. A., Ali, M. K., Alam, D., Yan, L. L., Levitt, N. S., Bernabe-Ortiz, A., et al. (2016). Obesity and its relation with diabetes and hypertension: A cross-sectional study across 4 geographical regions. *Global Heart*. 10.1016/j.gheart.2016.01.003 27102024 10.1016/j.gheart.2016.01.003 PMC4843822

[25] Verma, S., & Hussain, M. E. (2017). Obesity And diabetes: An update. *Diabetes Metab Syndr*. 10.1016/j.dsx.2016.06.017 27353549 10.1016/j.dsx.2016.06.017

[26] Stavropoulos-Kalinoglou, A., Metsios, G. S., Koutedakis, Y., & Kitas, G. D. (2011). Obesity in rheumatoid arthritis. *Rheumatology*. 10.1093/rheumatology/keq266 20959355 10.1093/rheumatology/keq266

[27] Kulkarni, K., Karssiens, T., Kumar, V., & Pandit, H. (2016). Obesity and osteoarthritis. *Maturitas*. 10.1016/j.maturitas.2016.04.006 27180156 10.1016/j.maturitas.2016.04.006

[28] Veenhof, C., Huisman, P. A., Barten, J. A., Takken, T., & Pisters, M. F. (2012). Factors associated with physical activity in patients with osteoarthritis of the hip or knee: A systematic review. *Osteoarthritis Cartilage*. 10.1016/j.joca.2011.10.016 22044842 10.1016/j.joca.2011.10.006

[29] Moreira, L. D., Oliveira, M. L., Lirani-Galvão, A. P., Marin-Mio, R. V., Santos, R. N., & Lazaretti-Castro, M. (2014). Physical exercise and osteoporosis: Effects of different types on bone and physical function in postmenopausal women. *Arquivos Brasileiros De Endocrinologia E Metabologia*. 10.1590/0004-2730000002913 25166042 10.1590/0004-2730000003374

[30] Llorente, I., García-Castañeda, N., Valero, C., González-Álvaro, I., & Castañeda, S. (2020). Osteoporosis in rheumatoid arthritis: Dangerous liaisons. Frontiers in Medicine. 10.3389/fmed.2020.601618 33330566 10.3389/fmed.2020.601618 PMC7719815

[31] Lane, N. E., Pressman, A. R., Star, V. L., Cummings, S. R., & Nevitt, M. C. (1995). Rheumatoid arthritis and bone mineral density in elderly women. *Journal of Bone and Mineral Research*. 10.1359/jbmr.1995.10.2.257 7754805 10.1002/jbmr.5650100212

[32] Dey, S., Eapen, M. S., Chia, C., Gaikwad, A. V., Wark, P. A. B., & Sohal, S. S. (2022). Pathogenesis and clinical features of asthma-COPD overlap. *American Journal of Physiology. Lung Cellular and Molecular Physiology*. 10.1152/ajplung.00121.2021 34668439 10.1152/ajplung.00121.2021

[33] Jenkins, C., FitzGerald, J. M., Martinez, F. J., Postma, D. S., Rennard, S., van der Molen, T., et al. (2019). Diagnosis and management of asthma, COPD and asthma-COPD overlap. *The Clinical Respiratory Journal*. 10.1111/crj.13016 30825365 10.1111/crj.13016

[34] White, V., Hill, D., Siahpush, M., & Bobevski, I. (2003). Trends in smoking prevalence among Australian adults from 1980 to 2001. *Tobacco Control*. 10.1136/tc.12.suppl_2.ii67scielo.br 12878776 10.1136/tc.12.suppl_2.ii67 PMC1766106

[35] Polosa, R., & Thomson, N. C. (2013). Smoking and asthma: Dangerous liaisons. *European Respiratory Journal*. 10.1183/09031936.00073312 22903959 10.1183/09031936.00073312

[36] Laniado-Laborín, R. (2009). Smoking and chronic obstructive pulmonary disease (COPD): Parallel epidemics of the 21st century. *International Journal of Environmental Research and Public Health*. 10.3390/ijerph6010209 19440278 10.3390/ijerph6010209 PMC2672326

[37] Australian Institute of Health and Welfare. Chronic obstructive pulmonary disease (COPD) [Internet]. Canberra: AIHW (2020). [cited 2025 Mar 20]. Available from: https://www.aihw.gov.au/reports/chronic-respiratory-conditions/copd

[38] Loza, E., Jover, J. A., Rodriguez, L., Carmona, L., & EPISER Study Group. (2009). Multimorbidity: prevalence, effect on quality of life and daily functioning, and variation of this effect when one condition is a rheumatic disease. *Seminars in Arthritis and Rheumatism*. 10.1016/j.semarthrit.2008.01.004 18336872 10.1016/j.semarthrit.2008.01.004

[39] Smith, A. W., Reeve, B. B., Bellizzi, K. M., Harlan, L. C., Klabunde, C. N., Amsellem, M., et al. (2008). Cancer, comorbidities, and health-related quality of life of older adults. *Health Care Financing Review*, *29*(4), 41–56. 18773613 PMC3142673

[40] Charles, S. T., & Carstensen, L. L. (2010). Social and emotional aging. Annual Review of Psychology. 10.1146/annurev.psych.093008.100448 19575618 10.1146/annurev.psych.093008.100448 PMC3950961

[41] Acree, L. S., Longfors, J., Fjeldstad, A. S., Fjeldstad, C., Schank, B., Nickel, K. J., et al. (2006). Physical activity and quality of life in older adults. *Health and Quality of Life Outcomes*. 10.1186/1477-7525-4-37 16813655 10.1186/1477-7525-4-37 PMC1524938

[42] Mitra, M., Chung, M. C., Wilber, N., & Walker, D. K. (2004). Smoking status and quality of life: A longitudinal study among adults with disabilities. *American Journal of Preventive Medicine*. 10.1016/j.amepre.2004.06.002 15450640 10.1016/j.amepre.2004.06.002

[43] Van den Bussche, H., Koller, D., Kolonko, T., Hansen, H., Wegscheider, K., Glaeske, G., et al. (2011). Chronic diseases and disease combinations in Multimorbidity in the elderly: cross-sectional study in Germany. *Bmc Public Health*. 10.1186/1471-2458-11-101 21320345 10.1186/1471-2458-11-101 PMC3050745

[44] Wong, A., Boshuizen, H. C., Schellevis, F. G., Kommer, G. J., & Polder, J. J. (2011). Examining Multimorbidity using longitudinal administrative data. *Journal of Clinical Epidemiology*. 10.1016/j.jclinepi.2010.12.011 21454049 10.1016/j.jclinepi.2010.12.011

[45] Haraldstad, K., Wahl, A., Andenæs, R., Andersen, J. R., Andersen, M. H., Beisland, E., et al. (2019). Quality of life research in medicine: A systematic review. *Quality of Life Research*. 10.1007/s11136-019-02214-9 31187410 10.1007/s11136-019-02214-9 PMC6761255

[46] Marengoni, A., Rizzuto, D., Wang, H. X., Winblad, B., & Fratiglioni, L. (2009). Patterns of chronic Multimorbidity in the elderly. *Journal of the American Geriatrics Society*. 10.1111/j.1532-5415.2008.02109.x 19207138 10.1111/j.1532-5415.2008.02109.x

[47] John, R., Kerby, D. S., & Hennessy, C. H. (2003). Comorbidity and Multimorbidity among American Indian elders. *The Gerontologist*. 10.1093/geront/43.5.649 14570961 10.1093/geront/43.5.649

[48] Islam, M. M., Valderas, J. M., Yen, L., Dawda, P., Jowsey, T., & McRae, I. S. (2014). Chronic disease Multimorbidity among senior australians: Prevalence and patterns. *PLoS One*. 10.1371/journal.pone.0083783 24421905 10.1371/journal.pone.0083783 PMC3885451

[49] Park, B., Lee, H. A., & Park, H. (2019). Identifying Multimorbidity patterns in Korean adults using latent class analysis. *PLoS One*. 10.1371/journal.pone.0216259 31721778 10.1371/journal.pone.0216259 PMC6853322

