# Supplementary material for: Comorbidity patterns and health-related quality of life in a cohort of Australian women cancer survivors
Source: Qual Life Res. 2026 Mar 1;35(4):84. doi: 10.1007/s11136-026-04191-2 (PMC12950653; doi:10.1007/s11136-026-04191-2)
Supplement: Supplementary file 1 — Supplementary Material 1 [file 11136_2026_4191_MOESM1_ESM.docx]

***Appendix***

Supplementary Figure 1. Derivation of the sample.
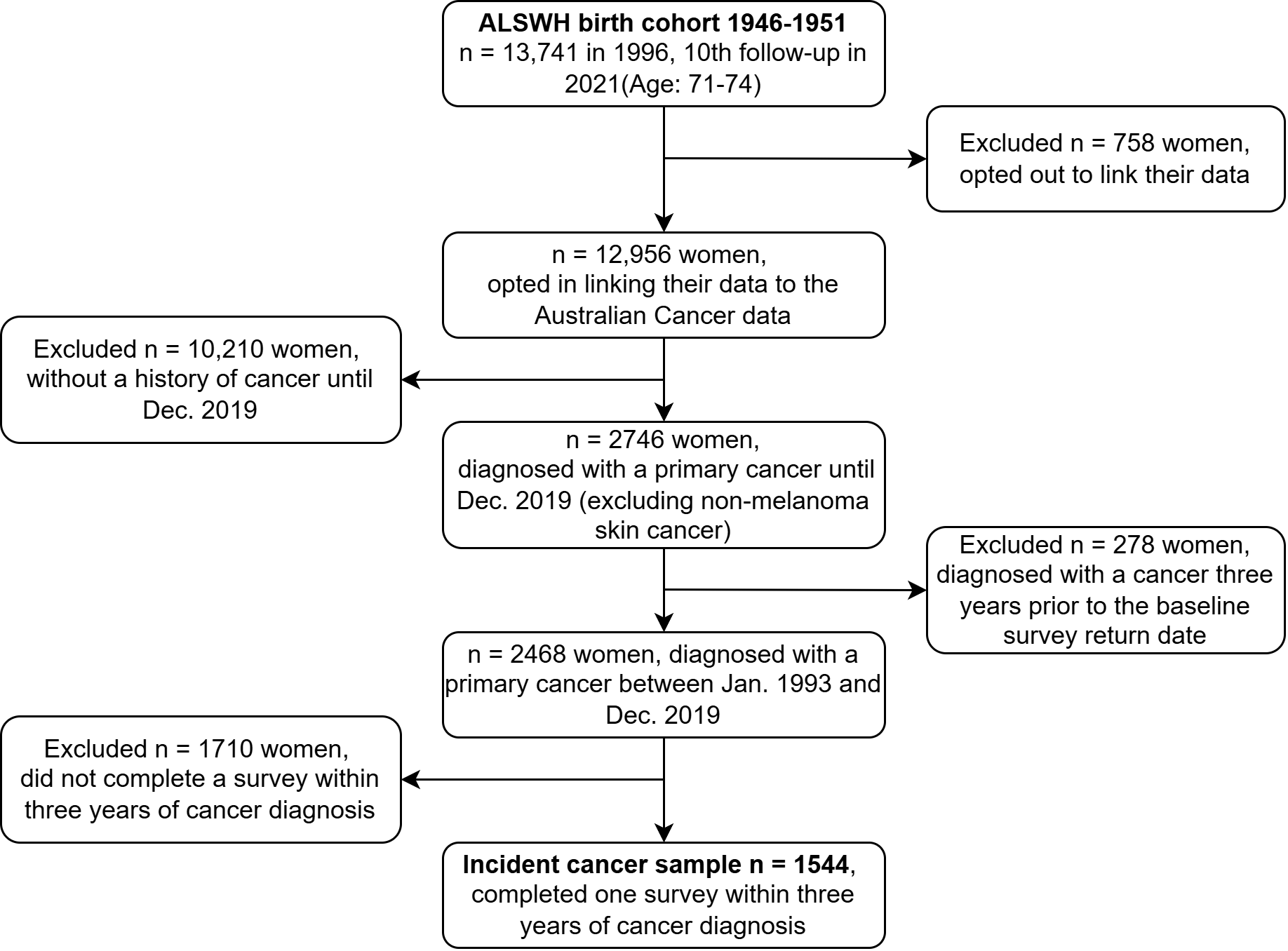


ALSWH: Australian Longitudinal Study on Women’s Health

| Supplementary Table 1. Classification of Cancer Sites and Corresponding ICD-O-3 Topography Codes | |
| --- | --- |
| Cancer site* | International Classification of Diseases for Oncology, 3rd Edition topography codes |
| Breast | C50.0 – C50.6, C50.8, C50.9 |
| Melanoma | C44.2 – C44.9 |
| Digestive | C18.0 - C18.3, C18.5 -C18.7, C18.9, C19, C20, C23, C21.0 C21.1, C21.8, C22.0, C22.7,  C24.1, C25.0 - C25.2, C25.4, C25.7, C25.9 |
| Gynaecological | C51.9, C53.0, C53.1, C53.9, C54.1, C54.3, C54.9, C55.9, C56.9, C57.0 |
| All other cancers | All other malignant neoplasms that were recorded in the ACD, and were diagnosed in our population after selection. |

* Excluding non-melanoma skin cancer that were not reported in the ACD. No additional cancer subtypes corresponding to the predefined categories were present among participants following selection. Cancer site categories were collapsed to protect confidentiality. ACD, Australian Cancer Database.

| Supplementary Table 2. Latent class analysis model fit statistics. | | | | |  |
| --- | --- | --- | --- | --- | --- |
| Number of classes (K) | Likelihood ratio (G^2^) | Degrees of freedom | AIC | BIC | Entropy |
| 2 | 503.2 | 1533 | 12331.6 | 12433.1 | 0.32 |
| 3 | 405.0 | 3066 | 12253.5 | **12408.4** | 0.35 |
| 4 | 364.7 | 4599 | 12233.2 | 12441.5 | 0.55 |
| **5** | 319.0 | 6132 | **12207.4** | 12469.2 | 0.62 |
| 6 | 302.7 | 7665 | 12211.2 | 12526.4 | 0.59 |
| 7 | 291.6 | 9198 | 12220.1 | 12588.7 | **0.67** |
| AIC, The Akaike information criterion; BIC, The Bayesian information criterion | | | | |  |

| Supplementary Table 3. Average posterior probability for the 5-class model. | | | |  |
| --- | --- | --- | --- | --- |
| Class number | Class label | Class size | Average Posterior Probability | |
| 1 | Relatively healthy | 880 | 0.82 | |
| 2 | Hypertension and arthritis | 247 | 0.61 | |
| 3 | Arthritis and osteoporosis | 135 | 0.75 | |
| 4 | Respiratory conditions | 169 | 0.74 | |
| 5 | Complex multimorbidity | 86 | 0.75 | |

| Supplementary Table 4. Membership Distributions in Percentages | | | | | | | |
| --- | --- | --- | --- | --- | --- | --- | --- |
| Number of Classes (k) | Class 1 | Class 2 | Class 3 | Class 4 | Class 5 | Class 6 | Class 7 |
| 2 | 66.0% | 34.0% | - | - | - | - | - |
| 3 | 66.2% | 10.9% | 22.9% | - | - | - | - |
| 4 | 12.3% | 6.0% | 32.7% | 49.0% | - | - | - |
| 5 | 58.0% | 16.3% | 8.9% | 11.1% | 5.7% | - | - |
| 6 | 17.5% | 6.9% | 13.1% | 11.1% | 2.4% | 48.9% | - |
| 7 | 2.0% | 10.2% | 16.7% | 7.5% | 14.1% | 44.0% | 5.7% |

**Latent Class Analysis Model Selection**

We fitted latent class models with two to seven classes. Model selection was guided by the Akaike Information Criterion (AIC), Bayesian Information Criterion (BIC), entropy, and substantive interpretability. The lowest AIC was observed for the five-class model, whereas the lowest BIC was observed for the three-class model; the highest entropy was observed for the seven-class model. The three-class model was excluded due to its low entropy, indicating substantial uncertainty in class membership assignment. The seven-class model was also excluded because of its poorer AIC and BIC values. In addition, this model identified a small class comprising only 2% of the population (n = 31), suggesting potential instability and limited clinical interpretability. Finally, given that the model included only nine indicator variables, a seven-class solution was considered overly complex.

| Supplementary Table 5. Statistical Tests of Inter-group Variable Distributions cross Latent Classes | | | | |
| --- | --- | --- | --- | --- |
| Variable Name | Test Type | Degrees of Freedom | Statistics | p value |
| **Age at cancer diagnosis** | Kruskal-Wallis | 4 | 237.34 | **< 0.0001** |
| Cancer site | χ^2^ | 16 | 17.08 | 0.3803 |
| **Marital status** | χ^2^ | 4 | 20.66 | **0.0004** |
| **Ability to manage available income** | χ^2^ | 8 | 50.01 | **< 0.0001** |
| Highest education | χ^2^ | 8 | 11.27 | 0.1868 |
| **Area of residence** | χ^2^ | 8 | 17.14 | **0.0287** |
| **Country of birth** | χ^2^ | 8 | 17.22 | **0.0279** |
| **Body mass index*** | χ^2^ | 8 | 117.52 | **< 0.0001** |
| **Smoking status** | χ^2^ | 8 | 31.28 | **0.0001** |

* Body mass index was categorised into three groups: underweight and normal weight (<25), overweight (≥25 and <30), and obese (≥30).

**Handling of Missing Data**

For variables that were expected to remain consistent over time. For example, *country of birth* is a fixed characteristic, and *area of residence* was assumed stable because the questionnaires were administered by post; any change of address would likely have resulted in loss to follow-up, reflecting a limitation of the cohort design beyond our control. *Highest education level* always captured the greatest level attained. Accordingly, we imputed missing information using either data from earlier waves prior to the index survey (for country of birth and area of residence) or from subsequent waves (for highest education level). We then examine the missingness patterns of the variables (Supplementary Table 5) and performed multiple imputation.

Multiple Imputation Strategy
Little’s MCAR test was conducted including all listed covariates in Supplementary Table 5, alongside HRQL domain scores, latent classes, and age at diagnosis [2]. The test failed to reject the null hypothesis, indicating no evidence against the assumption of data being Missing Completely at Random (MCAR). To address missing data, we applied multiple imputation by chained equations (MICE) [3], generating 20 imputed datasets [4]. The imputation model included marital status, ability to manage available income, highest education, area of residence, and country of birth. Each imputed dataset was analysed separately, and estimates were pooled using Rubin’s rules to incorporate both within- and between-imputation variability [5]. A sensitivity analysis comparing the imputed dataset with the complete-case analysis was performed. Results were identical to two decimal places; therefore, these findings are not presented.

| Supplementary Table 6. Missing observations in covariates used in multiple linear regression. | | | | |
| --- | --- | --- | --- | --- |
| Variable Name | Number (%) | Missing Pattern* | Fisher’s Exact Test ** | Little’s MCAR Test† |
| Age at cancer diagnosis | 0 | N/A | N/A | N/A |
| Marital status | 14 (0.01) | 7, 4, 2, 1, 0 | 0.64 | 0.33 |
| Ability to manage available income | 18 (0.01) | 13, 2, 1, 1, 1 | 0.86 | 0.59 |
| Highest education | 3 (0.00) | 3, 0, 0, 0, 0 | 1.00 | 0.69 |
| Area of residence | 2 (0.00) | 2, 0, 0, 0, 0 | 1.00 | 0.69 |
| Country of birth | 13 (0.00) | 7, 2, 3, 1, 0 | 0.47 | 0.42 |

MCAR = Missing completely at random.

* Missing pattern indicates the number of missing observations in each latent class. From left to right: class 1 *Relatively Healthy*, class 2 *Hypertension and arthritis*, class 3 *Arthritis and osteoporosis*, class 4 *Respiratory conditions*, class 5 *Complex multimorbidity*.

** Fisher’s exact test was used to examine the association between missingness of individual variables and latent class membership. [6]

† Little’s MCAR test was used to assess whether the overall pattern of missingness was consistent with MCAR, considering latent classes, health-related quality of life domain scores, and other covariates included in the multiple linear regression model.

**References:**

1. Kruskal WH, Wallis WA. Use of ranks in one-criterion variance analysis. J Am Stat Assoc. 1952;47(260):583-621. Available from: <https://doi.org/10.1080/01621459.1952.10483441>
2. Little RJA. A test of missing completely at random for multivariate data with missing values. *J Am Stat Assoc*. 1988;83(404):1198-202. Available from: <https://doi.org/10.1080/01621459.1988.10478722>
3. van Buuren S, Groothuis-Oudshoorn K. mice: Multivariate imputation by chained equations in R. *J Stat Softw*. 2011;45(3):1-67. Available from: <https://doi.org/10.18637/jss.v045.i03>
4. White IR, Royston P, Wood AM. Multiple imputation using chained equations: Issues and guidance for practice. *Stat Med*. 2011;30(4):377–99. Available from: <https://doi.org/10.1002/sim.4067>
5. Pearson K. On the criterion that a given system of deviations from the probable in the case of a correlated system of variables is such that it can be reasonably supposed to have arisen from random sampling. *Philos Mag*. 1900;50(302):157-75. Available from: <https://doi.org/10.1080/14786440009463897>
6. Fisher RA. On the interpretation of χ² from contingency tables, and the calculation of P. *J R Stat Soc*. 1922;85(1):87-94. Available from: <https://doi.org/10.2307/2340521>

Supplementary Figure 2. Distribution of health-related quality of life domain scores at baseline for the cohort population.


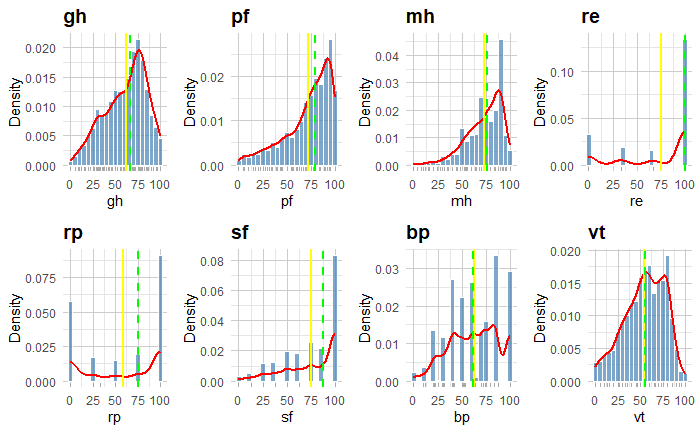


gh = general health; pf = physical functioning; mh = mental health; re = role emotional.

rp = role physical; sf = social functioning; bp = bodily pain; vt = vitality

Median = green dashed line; Mean = yellow solid line.


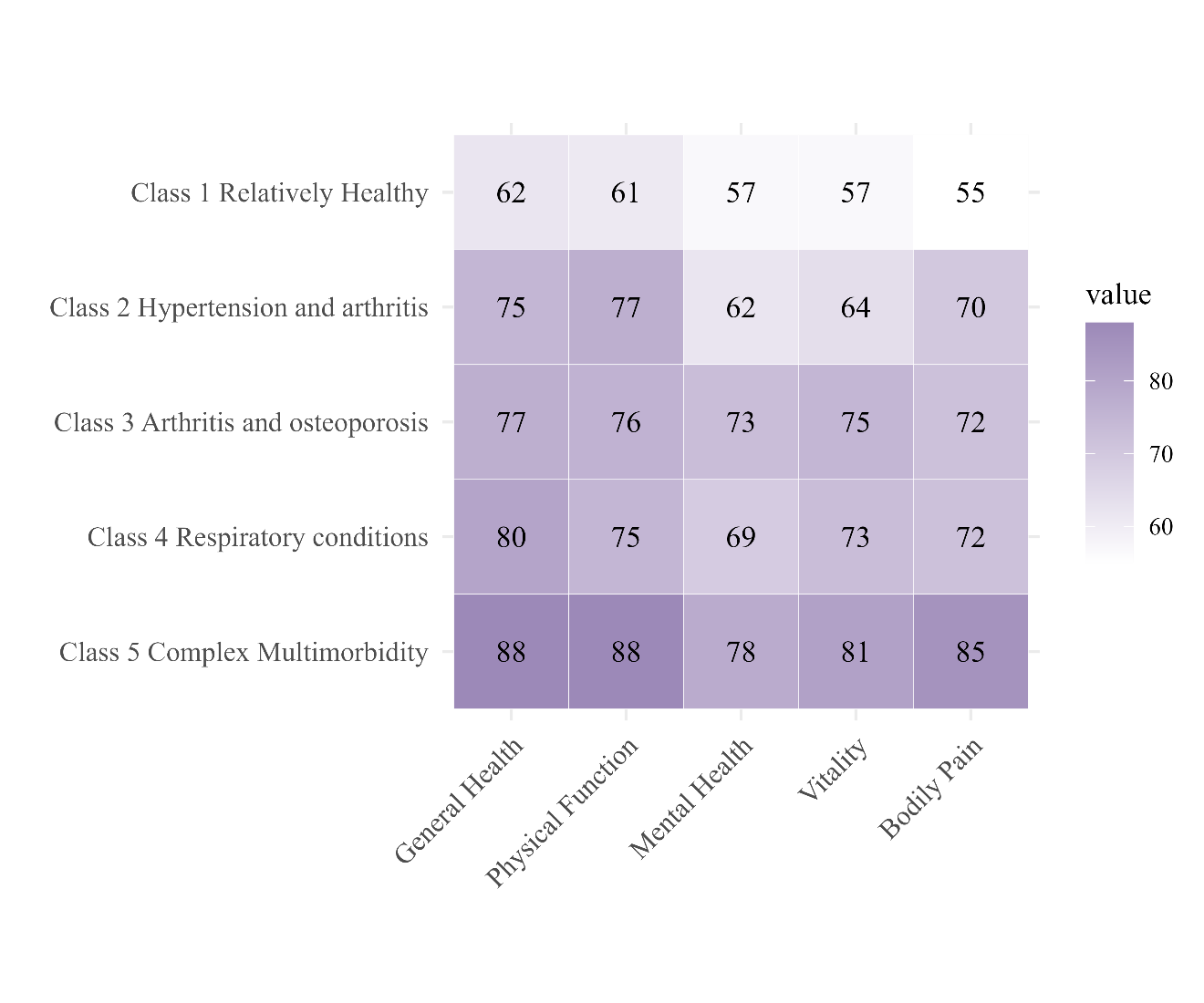
Supplementary Figure 3. Age-adjusted percentage of low health-related quality of life compared to population norm by latent classes.

* Numbers in each tile represents the percentage of the class who had a lower-than-median domain-specific HRQL score among all participants of her index survey. For example, if a woman was diagnosed with cancer before the fifth survey, her HRQL scores were compared to the population median of that follow-up.

Supplementary Table 6. Median and interquartile range of HRQL domains across ALSWH survey waves.

| Survey Wave | General Health | Physical Function | Mental Health | Vitality | Bodily Pain |
| --- | --- | --- | --- | --- | --- |
| 1* | 77 (62-87) | 90 (80-100) | 76 (64-88) | 60 (45-75) | 74 (51-84) |
| 2 | 77 (62-87) | 90 (80-95) | 80 (64-88) | 60 (45-75) | 74 (51-84) |
| 3 | 77 (62-87) | 90 (75-95) | 80 (64-88) | 60 (45-75) | 74 (51-84) |
| 4 | 77 (60-87) | 85 (75-95) | 80 (64-88) | 60 (45-75) | 72 (51-84) |
| 5 | 77 (62-87) | 85 (70-95) | 80 (64-88) | 65 (45-75) | 72 (51-84) |
| 6 | 72 (60-87) | 85 (70-95) | 80 (68-88) | 65 (45-80) | 72 (51-84) |
| 7 | 72 (62-87) | 85 (70-95) | 84 (68-92) | 65 (50-80) | 72 (51-84) |
| 8 | 72 (60-87) | 85 (65-95) | 84 (68-92) | 65 (50-80) | 72 (51-84) |
| 9 | 72 (57-85) | 80 (65-90) | 84 (68-92) | 65 (50-80) | 72 (51-84) |
| 10 | 72 (57-82) | 80 (60-90) | 80 (68-90) | 65 (50-75) | 62 (41-84) |
| HRQL = health-related quality of life; ALSWH = Australian Longitudinal Study on Women’s Health  * Baseline | | | | | |
